# Cost of vaccine delivery strategies in low- and middle-income countries during the COVID-19 pandemic

**DOI:** 10.1016/j.vaccine.2021.06.076

**Published:** 2021-08-16

**Authors:** Christina Banks, Allison Portnoy, Flavia Moi, Laura Boonstoppel, Logan Brenzel, Stephen C. Resch

**Affiliations:** a ThinkWell, Switzerland; b Center for Health Decision Science, Harvard T.H. Chan School of Public Health, United States; c Bill & Melinda Gates Foundation, United States

**Keywords:** Immunization, Cost, COVID-19

## Abstract

**Background:**

The COVID-19 pandemic has disrupted immunization services critical to the prevention of vaccine-preventable diseases in many low- and middle- income countries around the world. These services will need to be modified in order to minimize COVID-19 transmission and ensure the safety of health workers and the community. Additional budget will be required to implement these modifications that ensure safe delivery.

**Methods:**

Using a simple modeling analysis, we estimated the additional resource requirements associated with modifications to supplementary immunization activities (campaigns) and routine immunization services via fixed sites and outreach in 2020 US dollars. We considered the following four categories of costs: (1) personal protective equipment (PPE) & infection prevention and control (IPC) measures for immunization sessions; (2) physical distancing and screening during immunization sessions; (3) delivery strategy changes, such as changes in session sizes and frequency; and (4) other operational cost increases, including additional social mobilization, training, and hazard pay to compensate health workers.

**Results:**

We found that implementing a range of measures to protect health workers and communities from COVID-19 transmission could result in a per-facility start-up cost of $466–799 for routine fixed-site delivery and $12–220 for routine outreach delivery, and $12–108 per immunization campaign site. A recurrent monthly cost of $137–1,024 for fixed-site delivery and $152–848 for outreach delivery per facility could be incurred, and a $0.32–0.85 increase in the cost per dose during campaigns.

**Conclusions:**

By illustrating potential cost implications of providing immunization services through a range of strategies in a safe manner, these estimates can provide a benchmark for program managers and policy makers on the additional budget required. These findings can help country practitioners and global development partners planning the continuation of immunization services in the context of COVID-19.

## 1 Introduction

The COVID-19 pandemic has been disrupting immunization services that are critical to the prevention of morbidity and mortality from vaccine-preventable diseases in many low- and middle-income countries (LMICs) around the world [1]. Many LMICs have experienced drops in vaccination coverage due to interruptions and suspensions in the provision of essential routine services as well as decreases in the demand for those services due to concerns about contracting COVID-19 when interacting with the health care system [2], [3], [4]. Among the 73 countries historically receiving immunization support from Gavi, the Vaccine Alliance, 39 out of 68 planned vaccine introductions and campaigns that were projected to take place in 2020 have been delayed due to COVID-19 [5]. However, in these settings, the health benefits associated with routine childhood immunization greatly outweigh the COVID-19 related health risks [6], making it important to continue immunization services [7].

In order to maintain or restore immunization coverage levels while minimizing COVID-19 transmission and ensuring the safety of health workers and the community, modifications in the way vaccines are delivered are required. These modifications may change the cost of conducting immunization services, and may require additional budgetary support and planning to implement. World Health Organization (WHO) guidance advises countries to explore innovative methods for vaccine delivery to optimize service delivery while allowing for physical distance and minimizing the risks of COVID-19 transmission [8].

To help decision-makers in LMICs and global stakeholders understand the cost of such programmatic adaptations, we assessed the additional resource requirements associated with a number of modifications to various vaccine delivery strategies using a simple modeling analysis.

## 2 Methods

We analyzed the cost of four programmatic adaptations to illustrate the impact of global guidance and country practices on the cost of conducting immunization through routine fixed-site delivery, routine outreach, and campaigns. Two levels of intensity (low and high) were used to vary the measures for each cost category. WHO guidance provides a minimum standard for what is required to ensure the safe delivery of immunization services. The low-intensity scenarios and the cost assumptions were based on guidance and protocols from WHO on delivering immunization [8], [9], [10] and other essential health services in the context of COVID-19 [11], [12], [13], [14], [15], [16], [17], [18]. In practice, the way that modifications have been implemented differs across countries and has sometimes exceeded the set of minimum requirements recommended by WHO. The high-intensity scenarios were based on country policies and practices on delivering immunization services during COVID-19 and the Ebola epidemic.

Costs are reported as one-time investments and monthly costs that are assumed to recur as long as COVID-19 risk is present. Each cost category is presented as the incremental financial outlays required (i.e., financial costs) for the relevant adjustments. Costs are presented as per-dose estimates, and for routine fixed-site and outreach delivery, costs are also aggregated at the facility level for two facility types: low-volume and high-volume facilities.

### 2.1 Data sources

Country delivery cost data were extracted from studies published in the Immunization Delivery Cost Catalogue (IDCC) [19]: ten campaign studies [20], [21], [22], [23], [24], [25], [26], [27], [28], [29], 12 studies of new and underutilized vaccine introductions [23], [30], [31], [32], [33], [34], [35], [36], [37], [38], [39], [40], and primary data from two routine outreach studies [41], [42]. Data on service delivery logistics and the coverage levels achieved were extracted from the original studies and publications, and used to model potential changes in the cost of delivering immunization services on a per-dose or per-facility basis.

For routine fixed-site delivery, the number of health care workers, immunization sessions, and patients per session were simulated based on two typical facility profiles: a low-volume facility that organizes 1–2 immunization session per week, involving 2–4 health workers and immunizing 15 children per session; and a high-volume facility that organizes 5 sessions per week, involving a team of 2–4 health workers and administering doses to approximately 45 children each session (Appendix Table A). These facility profiles were based on the median sessions per week, health workers per session, and delivery volume from costing studies in Indonesia, Sierra Leone, Tanzania, and Vietnam [41], [42], [43], [44]. The cost of additional staff for fixed-site delivery was estimated by taking the average salary of the medical assistant cadre across all low- and lower middle-income countries in an adjusted ILO dataset [45]. Data on the operational costs of fixed-site delivery were extracted from the IDCC studies (Appendix Table B).

For campaign and outreach, all data were extracted from the studies, including the number of doses delivered, the number of vaccination team members, and salaries and per diem costs. From the campaign studies included for each analysis category, the median result is presented. Based on the distribution of volume delivered through outreach, Tanzania outcomes were considered as low-volume and Indonesia as high-volume facility examples.

Unit costs for personal protective equipment (PPE) and infection, prevention, and control (IPC) supplies and equipment were taken from WHO and UNICEF price lists, or from published literature [46], [47], [48], [49], [50]. All unit cost prices excluded the costs of insurance and freight and can be viewed in Appendix Table C. All results were converted to 2020 US dollars using World Bank official exchange rates [51] and IMF inflation rates (average consumer price) [52].

### 2.2 PPE & IPC measures for immunization sessions

The first cost category estimated the cost of providing health workers with PPE and offering hand hygiene facilities at vaccination sites. In the low-intensity scenario, medical masks were included for health workers, as recommended by the WHO in areas with known or suspected sporadic transmission of COVID-19 [8]. One mask was allocated per health worker per half day session to reflect that the mask should be replaced when damp.

The high-intensity scenario also included vaccinators wearing reusable goggles and changing gloves between beneficiaries, and non-vaccinator staff using one pair of gloves per half day session. The use of gloves during immunization sessions was reported in the DRC [53], India [54], Indonesia [55], Kenya [56], and Yemen [57]. The goggles were assumed to have a useful life of one year. For campaigns and outreach, one biohazard bag per team per day was allocated for the disposal of PPE supplies [8].

For all delivery strategies and scenario intensities, 12 ml of hand sanitizer per beneficiary was included. Two handwashing stations were added at outreach and campaign vaccination sites, to accommodate both the entry and exit points of each fixed outreach site, as seen during the measles outbreak response in Kinshasa in April 2020 [53]. The low-intensity scenario included simple handwashing stations, each consisting of a bucket with tap, while the high-intensity scenario included more advanced handwashing stations with a stand and basin.

### 2.3 Physical distancing and screening during immunization sessions

The second cost category included the cost of additional staff and supplies to conduct screening and ensure physical distancing during immunization sessions. One (low intensity) or two (high intensity) additional staff were added to campaign and outreach teams, as was the case during the measles campaign in Kinshasa in April 2020. To support routine immunization at fixed sites, we aligned additional staff assumptions based on the number of sessions per week in low-volume and high-volume facilities. We assumed 0.2 additional FTE for the low-intensity scenario and 0.4 additional FTE for the high-intensity scenario were allocated to each low-volume facility and we assumed 1 additional FTE for the low-intensity scenario and 1.4 additional FTE for the high-intensity scenario were allocated to each high-volume facility [53]. Additional campaign and outreach staff were assumed to be existing staff, and only received additional per diem payments, while the facility staff were allocated a medical assistant’s salary, equivalent to 56% of a nurse’s salary [45].

To better manage the flow of beneficiaries into and around health facilities, the routine fixed-site scenario included the provision of a roll of floor tape per week per facility for physical distance markings in both intensities and a screening tent per facility in the high-intensity scenario. The high-intensity scenario also included an infrared thermometer per vaccination team or facility for efficient screening of potential COVID-19 cases during immunization sessions, as listed in the priority medical devices list for the COVID-19 response as released by WHO [13], [47].

### 2.4 Delivery strategy changes

The third cost category estimated the cost of changes in daily targets, session sizes and frequency, focusing on routine outreach and campaign delivery. For campaign delivery, campaigns may reach fewer children per day, requiring more days to complete the campaign, which in turn would increase the per diem requirements for health workers, as well as any PPE and hand hygiene supplies. Reductions in daily coverage to 80% and 50% of the previous target were tested in the low- and high-intensity scenarios, respectively.

For routine outreach delivery, on the one hand, to reduce the number of touchpoints between health workers and the community, countries may choose to either: reduce the number of routine outreach sessions conducted; or, organize outreach sessions more frequently to ensure that session sizes stay small. The low-intensity scenario modelled the impact of halving the number of outreach sessions, while the high-intensity scenario modelled doubling the number of sessions. The number of children vaccinated in outreach overall was assumed to remain equal. These changes in session frequency had an impact on the per diem and transport costs.

On the other hand, to compensate for drops in attendance at routine facility- and school-based immunization sessions due to lockdown measures and community fear of COVID-19 transmission, countries may choose to increase outreach activities during or post lockdowns. In other words, the outreach efforts might be strategically scaled up to compensate for reductions in routine facility- and school-based delivery coverage. In addition to the previous approach regarding session frequency, we assumed intensified outreach to compensate for a 25% reduction in fixed-site coverage as a low-intensity scenario, and a 50% reduction in fixed-site coverage as a high-intensity scenario. These coverage drops are based on findings from a study on the impact of the pandemic on routine immunization in Karachi, Pakistan [3], and are in line with assumptions used in a modeling study of the effect of the pandemic on maternal and child mortality in LMICs [58]. The effect of increased outreach sessions to compensate for a drop in school-based coverage of 50% (low intensity) and 100% (high intensity) was also modelled for the high-volume facility setting.

### 2.5 Further operational cost increases

The fourth category assessed increases in other operational cost components that could reasonably be impacted by the COVID-19 pandemic. For example, WHO recommends that in order to sustain community demand for vaccination services, a tailored communication strategy should be implemented to provide accurate health information, address community concerns, enhance community linkages, and encourage continued use of immunization services [18].

For the routine program, potential cost increases of 50% (low intensity) or 100% (high intensity) of the mean training costs of a new vaccine introduction was assumed to cover the cost of ensuring physical distancing and other protective measures. Increases of 50% (low intensity) and 100% (high intensity) of mean new vaccine introduction costs for social mobilization were assumed to cover increased communication with the public regarding routine immunization services amidst the COVID-19 pandemic. The base level for training and social mobilization costs was based on 12 studies of new and underutilized vaccine introductions (Appendix Table B) [23], [30], [31], [32], [33], [34], [35], [36], [37], [38], [39], [40]. For campaigns, micro-planning, local transportation of staff and vaccines within the targeted area, social mobilization costs, supervision and vaccine storage fees were assumed to increase by 50–100%.

WHO recommends that renumeration should be commensurate with factors including additional hazards [59]. In order to reduce the risk of health worker absenteeism, costs for routine fixed-site vaccine delivery assumed the incentivization of health workers through providing hazard pay at 20% in the high-intensity scenario only [51]. Appendix Table D details the summary of assumptions for each presented cost category.

### 2.6 Full vaccination program case study in Tanzania

To illustrate the total programmatic cost of the adjustments needed to safely conduct immunization activities during the COVID-19 pandemic, we estimated total incremental costs of running the immunization program in Tanzania for one year (Appendix Table E). Using Tanzania-specific programmatic and costing data, the additional cost of implementing the previously described adjustments to maintain the routine vaccination schedule was assessed, as well as the additional cost of conducting a hypothetical nationwide measles follow-up or outbreak response campaign that would target all children aged 9 to 59 months old. The campaign cost assessment was based on a review of the IDCC and other forthcoming studies that report the unit cost of delivering measles, measles-rubella, meningitis A, and yellow fever vaccines through campaigns [21], [23], [35], [44], [60]. We then compared these costs to Tanzania’s annual spending on routine immunization [41].

## 3 Results

### 3.1 PPE & IPC measures for immunization sessions

For the low-intensity scenario, monthly PPE and IPC costs (masks and hand sanitizer) were estimated at $47–247 at fixed sites and $15–125 for outreach in low- and high-volume facilities, respectively, as shown in Table 1. In addition, the cost of handwashing stations for outreach sessions is estimated at $12–24 per facility, depending on the number of outreach teams that are simultaneously deployed. Per dose delivered, the increase in cost for fixed-site delivery is estimated at $0.20–0.33, an 11–19% increase compared with the pre-pandemic delivery cost per dose. When applying the low-intensity scenario PPE and IPC measures to campaigns, a 16% median increase in the recurrent cost per dose was seen.

**Table 1:** Cost increase from provision of PPE & IPC.

	Low-intensity scenario				High-intensity scenario			
**Routine delivery at low-volume facilities**	**One-time start-up cost per facility**	**Monthly cost per facility**	**Monthly cost per dose**	**Increase in cost per dose (%)**	**One-time start-up cost per facility**	**Monthly cost per facility**	**Monthly cost per dose**	**Increase in cost per dose (%)**
Fixed-site	0	47	0.20	11%	2	58	0.24	13%
Outreach	12	15	0.28	5%	84	19	0.36	7%

**Routine delivery at high-volume facilities**	**One-time start-up cost per facility**	**Monthly cost per facility**	**Monthly cost per dose**	**Increase in cost per dose (%)**	**One-time start-up cost per facility**	**Monthly cost per facility**	**Monthly cost per dose**	**Increase in cost per dose (%)**

Fixed-site	0	247	0.33	18%	4	297	0.40	22%
Outreach	24	125	0.26	19%	157	219	0.46	32%

**Campaigns**	**One-time start-up cost per site**	**Recurrent cost per dose**		**Increase in cost per dose (%)**	**One-time start-up cost per site**	**Recurrent cost per dose**		**Increase in cost per dose (%)**

	12	0.13		16%	82.97	0.31		39%

Note: IPC = infection prevention and control; PPE = personal protective equipment. Costs are presented in 2020 US dollars.

For the high-intensity scenario, in addition to the adjustments in the low-intensity scenario, gloves for all vaccination team members resulted in a total estimated monthly recurrent cost of $58–297 per facility for fixed-site delivery and $19–219 per facility for outreach. The one-time cost for reusable protective goggles and advanced handwashing stations at outreach sites amounted to $2–4 for fixed-site delivery and $84–157 for outreach. For campaigns, these measures resulted in an estimated 39% increase in the cost per dose.

For routine outreach delivery, masks accounted for the largest proportion of the recurrent cost in the low-intensity scenario (55–56%), while hand sanitizer was the biggest driver for campaigns and routine fixed-site delivery (62–85% and 52–70%, respectively). The greatest recurrent cost in the high-intensity scenario was gloves for routine outreach and campaigns, and hand sanitizer for routine fixed-site delivery. The upfront cost consisted entirely of the handwashing stations in the low-intensity scenario and the handwashing stations (95%) and reusable goggles in the high-intensity scenario (5%).

### 3.2 Physical distancing and screening during immunization sessions

For routine fixed-site delivery, Table 2 shows that the estimated results for mean monthly recurrent costs of hiring an additional staff member for 1 session per week at a low-volume facility to maintain physical distance and screening procedures were $84, or $90 when adding masks and tape. In the high-intensity scenario for low-volume facilities, the estimated mean monthly recurrent costs of hiring an additional staff member for 2 sessions per week were $169, as well as $3 for masks and $2 for gloves for the additional staff member and an additional $4 for tape. In the low-intensity scenario at high-volume facilities, there was a mean monthly recurrent cost of $422 for 1 additional FTE (i.e., a full-time staff member), as well as $7 for masks for gloves for the additional staff member and an additional $4 for tape. In the high-intensity scenario at high-volume facilities, there was a mean monthly recurrent cost of $591 for 1.4 additional FTE, as well as $10 for masks and $8 for gloves and an additional $4 for tape. Additionally, in the high-intensity scenario for both low- and high-volume facilities, we assumed one-time start-up costs of $25 per thermometer and $150 per tent per facility for screening measures. Overall and on average, we estimated a $90 increase per low-volume facility and a $433 increase per high-volume facility in the low-intensity scenario and $353 per low-volume facility ($178 recurrent, $175 start-up) and $788 per high-volume facility ($613 recurrent, $175 start-up) in the high-intensity scenario for physical distance and patient screening.

**Table 2:** Cost increase from implementing social distancing and screening measures during immunization sessions.

	Low-intensity scenario				High-intensity scenario			
**Routine delivery at low-volume facilities**	**One-time start-up cost per facility**	**Monthly cost per facility**	**Monthly cost per dose**	**Increase in cost per dose (%)**	**One-time start-up cost per facility**	**Monthly cost per facility**	**Monthly cost per dose**	**Increase in cost per dose (%)**
Fixed-site	0	90	0.38	20%	175	178	0.75	40%
Outreach delivery	0	19	0.48	9%	25	40	1.01	20%

**Routine delivery at high-volume facilities**	**One-time start-up cost per facility**	**Monthly cost per facility**	**Monthly cost per dose**	**Increase in cost per dose (%)**	**One-time start-up cost per facility**	**Monthly cost per facility**	**Monthly cost per dose**	**Increase in cost per dose (%)**

Fixed-site	0	433	0.59	31%	175	613	0.83	45%
Outreach delivery	0	24	0.05	4%	47	105	0.24	17%

**Campaign**	**One-time start-up cost per site**	**Recurrent cost per dose**		**Increase in cost per dose (%)**	**One-time start-up cost per site**	**Recurrent cost per dose**		**Increase in cost per dose (%)**

	0.00	0.08		10%	25.04	0.17		20%

Note: Costs are presented in 2020 US dollars.

For outreach, mean monthly recurrent costs for additional distancing and screening measures amounted to $19–24 (low intensity) and $40–105 (high intensity), as well as $25–47 of upfront costs per facility. In the low-volume delivery setting where almost all workers conducting outreach receive per diems, these accounted for 84–87% of the costs between the two intensities, with the remainder being masks and gloves.

For campaigns, a 10% and 20% increase in the recurrent cost per dose were seen when applying the low- and high-intensity scenarios respectively. In both intensities, per diems for the additional crowd controllers were the major cost driver, accounting for upwards of 92% of costs in the low-intensity scenario and 89% in the high-intensity scenario. PPE for the crowd controllers covered the remainder.

### 3.3 Delivery strategy changes

Doubling the frequency of outreach sessions resulted in an increased monthly recurrent cost of $79 according to the cost profile of Tanzania, where facilities delivered 1–41% through outreach, and $91 according to the cost profile of Indonesia, where facilities delivered 39–74% through outreach. The monthly recurrent cost consisted mostly of per diem payments in low-volume facilities (84%) and transport costs in high-volume facilities (97%), only 5/24 of which paid per diems for outreach activities. Halving the number of outreach sessions did not generate additional costs, and in our model would technically result in cost savings if health workers would receive less per diems for outreach as a result.

A 25% increase in the number of outreach beneficiaries to compensate for a fall in fixed-site coverage led to a $2–238 monthly increase in transport and per diem costs (low intensity). Table 3 shows that when outreach beneficiaries were increased by 50% in the high intensity, a $5–475 monthly recurrent cost was incurred. The effect was not strong in settings with high-volume facilities which already delivered most outreach doses in a large number of low-volume sessions; fewer facilities in these settings provided per diems for outreach activities which further contributed to the lower costs generated.

**Table 3:** The monthly cost per facility incurred from increasing the number of outreach sessions to compensate for reductions in fixed-site delivery.

	Monthly cost per facility	
	Low-intensity scenario	High-intensity scenario
Tanzania: Increase outreach beneficiaries	238	475
Indonesia: Increase outreach beneficiaries (reduction in fixed-site delivery)	2	5
Indonesia: Increase outreach beneficiaries (reduction in school-based delivery)	14	28

Note: In Tanzania, facilities delivered 1–41% through outreach. In Indonesia, facilities delivered 39–74% through outreach and 14%–33% at schools. Costs are presented in 2020 US dollars.

Reducing daily campaign coverage to 80% of previous levels and extending the campaign to cover the same number of beneficiaries results in a 9% increase in the cost per dose ($0.06). This rises to a 35% increase in cost per dose ($0.25) with a fall in daily coverage to 50%. The additional costs are from per diems for health workers.

### 3.4 Further operational cost increases

For routine fixed-site delivery, we estimated the mean monthly recurrent costs of hazard pay per facility to be $84 in the high-intensity scenario, as can be seen in Table 4. The one-time start-up costs per facility for training was $241 in the low-intensity scenario and $322 in the high-intensity scenario on average. The one-time start-up costs per facility for social mobilization was $224 in the low-intensity scenario and $299 in the high-intensity scenario on average. The low-intensity scenario did not include any monthly recurrent costs, and neither scenario included monthly recurrent costs for social mobilization and training.

**Table 4:** The impact of operational cost increases for facility-based routine delivery and campaigns.

**Routine fixed-site delivery at low-volume facilities**	**One-time start-up cost per facility**		**One-time start-up cost per facility**	**Monthly cost per facility**	**Monthly cost per dose**	**Increase in cost per dose (%)**
Hazard pay	0		0	84	0.36	19%
Social mobilization	224		224	0	0	0%
Training	241		241	0	0	0%

**Routine fixed-site delivery at high-volume facilities**	**One-time start-up cost per facility**		**One-time start-up cost per facility**	**Monthly cost per facility**	**Monthly cost per dose**	**Increase in cost per dose (%)**

Hazard pay	0		0	84	0.12	6%
Social mobilization	299		299	0	0	0%
Training	322		322	0	0	0%

**Campaign**	**Recurrent cost per dose**	**Increase in cost per dose (%)**	**Recurrent cost per dose**			**Increase in cost per dose (%)**

	0.23	20%	0.45			40%

Note: Costs are presented in 2020 US dollars.

If campaign expenses on social mobilization activities, training, transport, communication and microplanning were to increase by 50% (low intensity), the cost of the campaign would increase by 20% or approximately 40% if they were to double.

### 3.5 Total costs, including all cost categories

If all low-intensity measures were to be implemented simultaneously, this would result in a $466 start-up cost (at both low- and high-volume facilities) and $137–680 monthly recurrent cost per facility for fixed-site delivery on average, as shown in Table 5. Recurrent costs in low-volume facilities largely consisted of the additional crowd controller cost (53–62%), while at high-volume facilities, these costs accounted for 58–62%. Start-up costs in low-volume facilities are almost entirely from the added social mobilization and training. Combining the high-intensity measures for fixed site delivery results in an average start-up cost of $798–799 and a mean monthly recurrent cost of $317–1,024. The average start-up cost per facility of implementing all the low-intensity measures for routine outreach is $12–24 compared to $122–220 for the high-intensity measures, consisting largely of the handwashing station costs. The mean monthly recurrent cost per facility was found to be $152–332 for the low-intensity measures combined and $333–848 for the high-intensity measures. For campaigns, the total start-up cost per site ranged from $12–108 depending on intensity and an increase in the recurrent cost per dose of $0.32–0.85, up to 144% higher than without any measures. The increase in the low-intensity recurrent cost per dose was driven by the additional IPC measures (35%) and operational cost increases (31%), while the largest cost driver for the high-intensity recurrent cost per dose increase was the PPE items (27%), followed by the operational cost increases (24%).

**Table 5:** The total cost of all measures being implemented.

	Low-intensity scenario				High-intensity scenario			
**Routine delivery at low-volume facilities**	**One-time start-up cost per facility**	**Monthly cost per facility**	**Monthly cost per dose**	**Increase in cost per dose (%)**	**One-time start-up cost per facility**	**Monthly cost per facility**	**Monthly cost per dose**	**Increase in cost per dose (%)**
Routine fixed-site delivery	466	137	0.58	31%	798	317	1.35	72%
Routine outreach delivery	12	329	5.05	99%	122	848	12.84	251%

**Routine delivery at high-volume facilities**	**One-time start-up cost per facility**	**Monthly cost per facility**	**Monthly cost per dose**	**Increase in cost per dose (%)**	**One-time start-up cost per facility**	**Monthly cost per facility**	**Monthly cost per dose**	**Increase in cost per dose (%)**

Routine fixed-site delivery	466	680	0.92	49%	799	1,024	1.39	74%
Routine outreach delivery	24	152	0.33	23%	220	333	0.72	51%

**Campaign**	**One-time start-up cost per site**	**Recurrent cost per dose**		**Increase in cost per dose (%)**	**One-time start-up cost per site**	**Recurrent cost per dose**		**Increase in cost per dose (%)**

	12	0.32		55%	108	0.85		144%

Note: Costs are presented in 2020 US dollars.

### 3.6 Vaccination program case study in Tanzania

We applied the assumptions and parameters discussed above to data from a recent study conducted in Tanzania on the cost of vaccine delivery as an illustration. Implementing all the proposed adjustments to safely conduct immunization activities during the COVID-19 pandemic across all facilities offering routine immunization services in Tanzania would result in an incremental annual cost of $19.0 million to $123 million, depending on the scenario intensity. This represents an overall cost increase of 89–147% from a baseline annual EPI total cost of $138 million [41]. Incremental program costs for the low- and high-intensity scenarios, disaggregated by cost item, are presented in Table 6. The change in recurrent cost for halving outreach sessions in the low-intensity scenario was capped at $0 as our model did not consider the possibility of cost savings arising.

**Table 6:** Incremental vaccination program costs in Tanzania.

	Low-intensity scenario		High-intensity scenario	
	Fixed/upfront	Recurrent	Fixed/upfront	Recurrent
PPE costs	0	5,630,000	10,600	20,200,000
IPC costs	364,000	2,170,000	2,320,000	2,170,000
Physical distancing and screening costs	342,000	5,570,000	1,490,000	25,800,000
Outreach session frequency change costs	0	0	0	53,100,000
Hazard pay costs	0	0	0	8,400,000
Operational costs (trainings, social mobilization)	0	4,880,000	0	9,770,000
Total costs	707,000	18,300,000	3,820,000	119,000,000

Note: IPC = infection prevention and control; PPE = personal protective equipment. Costs are presented in 2020 US dollars.

In the low-intensity scenario, fixed upfront costs represent an increase of $707,000 (0.5% of baseline program costs). These costs are split evenly between IPC and distancing and screening costs and include costs to provide basic handwashing stations to teams conducting routine outreach immunization services, as well as tape to facilitate physical distancing during facility-based immunization sessions. For the low-intensity scenario, recurrent annual costs are estimated to be $18.3 million (a 13% increase from baseline). This includes costs of providing PPE for all workers, soap and hand sanitizer, as well as a moderate increase in training and social mobilization costs. In the low-intensity scenario, the biggest costs drivers of recurrent costs are PPE costs and distancing and screening costs, each contributing about 30% of the total increase in recurrent costs.

In the high-intensity scenario, fixed upfront costs amount to an increase of $3.8 million (or 3% of baseline costs). Additional one-off costs for this scenario intensity include providing a tent and an infrared thermometer for screening at each health facility, as well as protective goggles for vaccinators, and an advanced handwashing station for outreach teams. Recurrent costs for the high-intensity scenario represent an increase of $119 million (86% of baseline program costs). Among these costs, a doubling in the frequency of outreach sessions represents the biggest cost driver (44% of incremental recurrent costs), followed by the additional costs to enforce distancing and screening measures (22% of recurrent costs) and PPE costs (17% of recurrent costs).

Implementing all the adjustments in a nation-wide measles campaign with a target population of 9,738,602 children [61] and a coverage level of 88% [62] would lead to an incremental cost of $0.38 to $1.35 per dose, respectively, in the low-intensity and high-intensity scenarios, up from a financial cost per dose estimate of $0.43 [21], [23], [35], [44], [60]. The additional low- and high-intensity scenario costs increase the financial cost per dose by between 112% and 315%. Therefore, the incremental cost of COVID-19 related measures would have a large impact on the financial cost of the campaign.

## 4 Discussion

This study found that implementing a range of measures to protect health workers and communities from COVID-19 transmission could result in a per-facility start-up cost of $466–799 for routine fixed-site delivery and $12–220 for routine outreach delivery, and $12–108 per immunization campaign site. A recurrent monthly cost of $137–1,024 for fixed-site delivery and $152–848 for outreach delivery per facility could be incurred, and a $0.32–0.85 increase in the cost per dose during campaigns. We also presented a case study in Tanzania in order to provide a real-world example of the cost implications investigated in our analysis; however, alternative country settings may have different cost implications compared to this case study. For example, the estimated cost increase of 89–147% is based on the annual program cost of $138 million in Tanzania, but we might expect cost increases to vary according to the annual program cost.

Several modeling studies have shown the detrimental impact that essential health service disruptions could have on the burden of vaccine-preventable diseases [8], [48] and others such as HIV [63], TB [64], and malaria [65]. The WHO has previously estimated the costs of maintaining essential health services for a group of low- and middle-income countries to estimate global level financial implications [8], [48]. Our study fills a gap in knowledge of the financial implications by estimating the financial costs specific to maintaining immunization services at the country level. The various scenarios and cost estimates per country, per facility, and per dose are aimed to facilitate discussions at the country level and inform country planning and resource mobilization efforts.

Some of the scenarios presented included more PPE than is required as per WHO’s minimum guidance. WHO guidance indicates that vaccinators could consider extended use of masks in areas with widespread transmission, that masks are not needed for screening, and that vaccinators do not need to wear gloves if the skin is intact [66]. Nevertheless, several countries have offered vaccinators with PPE above WHO recommendations during the COVID-19 pandemic, for example, wearing gloves during immunization. Studies of the Ebola epidemic demonstrate the impact of a breakdown in trust between communities and the health system, as well as health worker absenteeism due to sickness or fear of being sick [67], [68]. Our results signal that the additional costs of PPE are not a large cost driver compared to other interventions. Arguably, a conservative package of PPE supply may prevent the need for much costlier interventions later on to restore coverage levels and rebuild trust in the health system.

The Ebola epidemic and its devastating impact on the immunization program and broader health system are well-documented, providing valuable programmatic lessons [67], [68], [69], [70], [71]. However, cost evidence around the impact and the cost of maintaining and restoring immunization is limited. Because of the paucity of cost evidence, our analysis could only explore broad ranges on potential scenarios for the additional costs of components such as social mobilization and hazard pay. There remains uncertainty around these additional costs. There may also be the need to hire additional staff (surge) to support vaccine delivery needs. Our estimates included the potential for hiring workers at the level of a community health worker or nursing assistant that could represent an upper bound of what might be required. More work is needed to not only systematically document country experiences during the COVID-19 pandemic, but also to analyze the actual delivery costs and cost-effectiveness of interventions that are identified as best practices.

The results of this analysis are meant to offer general guidance but should be interpreted with caution, as several limitations apply. First, the analysis relied on the data as reported by the authors, and several assumptions had to be made in the classification of certain costs, in the estimations of salaries, etc. We also relied on evidence from four country costing studies to develop our low- and high-volume profiles for facilities [41], [42], [43], [44], but changes to session size, number of health workers per session, and delivery volume could have different cost implications at the per-facility and per-dose level. Second, some of the prices for COVID-19 response materials have changed rapidly, and further changes may affect the accuracy of the results over time. Our estimates did not factor in the costs of locally sourced or produced PPE and IPC materials given the heterogeneity that would have been introduced into this aggregate model. However, our analysis relying on global price lists could be considered an average for these estimates. Likewise, we did not include shipping costs of PPE and IPC materials, due to the lack of reliable estimates and approaches for country-level sourcing of PPE. Third, the assumptions for PPE used are based on current global guidance and country practice, but we did not consider the additional cost of PPE due to leakages, wastage, or other forms of PPE contamination. Fourth, our model also assumed overall immunization coverage across all services remains equal under all circumstances, regardless of the delivery strategy mix or any supply or demand disruptions that were not included in the model. Fifth, we have not taken into account the changes to shared costs and potential efficiencies gained from more frequent cleaning and sanitization of surfaces, combining multiple well-child services to minimize the number of visits needed per child, changes to scheduling to spread immunization sessions throughout the day, and delivery of well-child services within the facility that is physically separate from areas providing services to sick patients. Overall, country-specific guidance and policies should be reviewed before translating these results to other country contexts, as well as changes due to delivery of the COVID-19 vaccine itself. As we learn more about the effectiveness of the COVID-19 vaccine in different settings and particularly questions of whether individuals who are vaccinated can still transmit infection to others, the recommendations for PPE/IPC may change, which would likewise change the cost implications. These mitigation strategies also may be time-limited in low- and middle-income countries once healthcare workers are vaccinated and/or the general population is adequately vaccinated. Despite the estimated potential increases in the costs of delivering vaccines during the COVID-19 pandemic, the cost-effectiveness of vaccine programs is likely to remain favorable [6], particularly in settings where COVID-19 vaccine coverage is low.

Finally, we are comparing cost implications for routine fixed facility, routine outreach, and mass vaccination campaign delivery strategies. However, each delivery strategy relies on a different set of studies, and the underlying group of countries and studies between strategies are not perfectly comparable. In addition, there are many additional delivery modalities, delivery strategies, antigens, and country settings that would have different cost implications that have not been included in this analysis. Furthermore, there is methodological heterogeneity within the studies for each strategy.

In order to improve cost estimates for strategic planning in the COVID-19 pandemic, improved documenting of the country-level costs of conducting immunization services during the COVID-19 pandemic may be required. These analyses are meant to illustrate potential cost implications from providing immunization services through a range of strategies in a safe manner, based on global guidance and country protocols. These estimates can provide a benchmark for program managers and policy makers on the additional budget required. The findings can help country practitioners and global development partners in the design and implementation of strategies to ensure the continuation of immunization services in the context of COVID-19.

## 5 Contributors

CB, LBo, FM, AP, SCR carried out the analysis. CB, LBo, FM, AP drafted the initial manuscript. All authors conceptualized and designed the study, critically reviewed the analysis, revised the manuscript, and approved the final manuscript as submitted.

## Declaration of Competing Interest

The authors declare the following financial interests/personal relationships which may be considered as potential competing interests: [LBr was employed by the Bill & Melinda Gates Foundation while contributing to the study. The other authors declare no competing interests.].
