# Supplementary material for: Cost of vaccine delivery strategies in low- and middle-income countries during the COVID-19 pandemic
Source: Vaccine. 2021 Aug 16;39(35):5046–54. doi: 10.1016/j.vaccine.2021.06.076 (PMC8238647; doi:10.1016/j.vaccine.2021.06.076)
Supplement: Supplementary data 1 [file mmc1.docx]

**Supplementary Appendix**

**Table A. Assumptions for fixed-site calculations and descriptive information of outreach data from country studies.**

| **Routine delivery at fixed-sites** | Session size | Session frequency (per week) | Staff per team |
| --- | --- | --- | --- |
| Low-volume | 15 | 1–2 | 2–4 |
| High-volume | 45 | 5 | 2–4 |
| **Routine delivery through outreach** | Output through outreach per month (median) | Session frequency (per month, median) | Staff per outreach team (median) |
| Low-volume (Tanzania) [41] | 38 | 2 | 2 |
| High-volume (Indonesia) [42] | 351 | 24 | 4 |

**Table B. Per-facility social mobilization and training costs for routine fixed sites extracted from Immunization Delivery Cost Catalogue studies (2020 US dollars).**

| **Reference** | **Country** | **Year** | **Social mobilization** | **Training** |  |
| --- | --- | --- | --- | --- | --- |
|  |  |  |  |  |  |
| Kaucley 2015 [23] | Benin | 2011 | $35 | $43 |  |
| Ruhago 2015 [38] | Tanzania | 2012 | $606 | $231 |  |
| Castañeda-Orjuela 2013 [33] | Colombia | 2009 | $269 | NA |  |
| Douba 2011 [35] | Côte d'Ivoire | 2006 | $329 | $1,436 |  |
| Griffiths 2005 [36] | Mozambique | 2001 | $4 | $29 |  |
| PAHO 2014 [32] | Honduras | 2011 | $140 | $17 |  |
| Gotsadze 2014 [30] | Moldova | 2011 | $842 | $260 |  |
| Dorji 2018 [34] | Bhutan | 2017 | $55 | $317 |  |
| Yin 2012 [40] | China | 2009 | $76 | $120 |  |
| Usuf 2014 [39] | Gambia | 2009 | $310 | $161 |  |
| Guthrie 2014 [31] | Uganda | 2011 | $159 | $527 |  |
| Ngabo 2015 [37] | Rwanda | 2012 | $84 | $59 |  |

Note: Per-facility costs extrapolated to average for low intensity (50% increase) and high intensity (100% increase) of reported values (rounded to the nearest ten).

**Table C. Price Assumptions**

| **Item** | **Unit cost (USD 2020)** | **Source** |
| --- | --- | --- |
| 1 mask | $ 0.31 | WHO Emergency Global Supplies Catalogue^1^ |
| 1 set of examination gloves | $ 0.18 | WHO Emergency Global Supplies Catalogue^1^ |
| 1 pair of goggles | $ 1.83 | WHO Emergency Global Supplies Catalogue^1^ |
| 1 biohazard bag | $ 0.17 | WHO Emergency Global Supplies Catalogue^1^ |
| 1 unit of soap (1 l.) | $ 0.90 | WHO Essential Supplies Forecasting Tool V2^2^ |
| 1 60-liter bucket | $ 6.23 | Freedman et al.^3^ |
| 1 stand | $ 31.15 | Freedman et al.^3^ |
| 1 basin | $ 2.27 | Freedman et al.^3^ |
| Infrared thermometer | $ 25.04 | UNICEF supply catalogue^4^ |
| Hand sanitizer (1 l.) | $ 8.30 | WHO Essential Supplies Forecasting Tool V2^2^ |
| 1 roll of tape | $1.00 | Assumption |
| 1 screening tent | $150.00 | GEVIT Vaccination Strategies Forecasting Tool^5^ |

**References**

1. World Health Organization. Emergency Global Supply Chain System (COVID-19) Catalogue. 18 November 2020. [Online] Accessed 4 December 2020. Available at: https://www.who.int/publications-detail-redirect/emergency-global-supply-chain-system-(covid-19)-catalogue

2. World Health Organization. WHO COVID-19 Essential Supplies Forecasting Tool (ESFT). 29 April 2020. [Online] Accessed 14 July 2020. Available at: https://www.who.int/docs/default-source/coronaviruse/covid-esft-v2-who-release-updated20200429-1650edt.xlsx?sfvrsn=6b46f7b0_2&download=true

3. Freedman M, Bennett SD, Rainey R, Otieno R, Quick R. Cost analysis of the implementation of portable handwashing and drinking water stations in rural Kenyan health facilities. *Journal of water, sanitation, and hygiene for development.* 2017;7(4):659-664.

4. UNICEF Supply Division. Supply Catalogue: Unit price for an infrared clinical thermometer. Accessed 14 July 2020. [Online] Available at: https://supply.unicef.org/s0481054.html.

5. World Health Organization. Global Ebola Vaccine Implementation Team (GEVIT) Practical Guidance on the Use of Ebola Vaccine in an outbreak response. Appendix K accination Strategies Forecasting Tool. May 2016. [Online] Accessed 14 July 2020. Available at: https://www.who.int/csr/resources/publications/ebola/gevit-guide/en/.

**Table D. Summary of assumptions by cost category.**

**Scenario 1: Personal protective equipment (PPE) & infection prevention and control (IPC).**

| **Strategy** | **Low-intensity scenario** | **High-intensity scenario** |
| --- | --- | --- |
| Campaign | **PPE**   - 1 medical mask per health worker per half day session (2 per full day session) - 1 biohazard waste bag per session/team   **IPC**   - Two simple hand washing stations for each fixed vaccination post (1 x 60 liter bucket each) - 12ml of soap per beneficiary - 12ml of hand sanitizer per beneficiary - 1 L water per beneficiary | **PPE**   - 1 medical mask per health worker per half day session (2 per full day session) - 1 biohazard waste bag per session/team - Vaccinators receive one set of reusable goggles, useful life of 1 year - 1 pair of gloves used per beneficiary - 1 pair of gloves for non-vaccinator health workers per half day session (2 per full day session)   **IPC**   - Two advanced hand washing station for each vaccination post (1 x 60 liter bucket, 1 x stand, 1 x basin each) - 12ml of soap per beneficiary - 12ml of hand sanitizer per beneficiary - 1 L water per beneficiary |
| Routine outreach | **PPE**   - 1 medical mask per health worker per half day session (2 per full day session) - 1 biohazard waste bag per session/team   **IPC**   - Simple hand washing station for each fixed vaccination post (2 x 60 liter bucket) - 12ml of soap per beneficiary - 12ml of hand sanitizer per beneficiary - 1 L water per beneficiary | **PPE**   - 1 medical mask per health worker per half day session (2 per full day session) - 1 biohazard waste bag per session/team - Vaccinators receive one set of reusable goggles, useful life of 1 year - 1 pair of gloves used per beneficiary - 1 pair of gloves for non-vaccinator health workers per half day session (2 per full day session)   **IPC**   - Advanced hand washing station for each vaccination post (2 x 60 liter bucket, 2 x stand, 2 x basin) - 12ml of soap per beneficiary - 12ml of hand sanitizer per beneficiary - 1 L water per beneficiary |
| Routine facility-based | **PPE**   - 1 medical mask per health worker per half day session (2 per full day session)   **IPC**   - 12ml of hand sanitizer per beneficiary | **PPE**   - 1 medical mask per health worker per half day session (2 per full day session) - Vaccinators receive one set of reusable goggles, useful life of 1 year - 1 pair of gloves used per beneficiary - 1 pair of gloves for non-vaccinator health workers per half day session (2 per full day session)   **IPC**   - 12ml of hand sanitizer per beneficiary |

**Scenario 2: Physical distancing and screening.**

| **Strategy** | **Low-intensity scenario** | **High-intensity scenario** |
| --- | --- | --- |
| Campaign | **Additional personnel**   - 1 additional member of staff per vaccination team   **Additional PPE**   - 1 medical mask per additional staff member per half day session (2 per full day session)   **Physical distancing supplies**   - None   **Screening equipment**   - None | **Additional personnel**   - 2 additional members of staff per vaccination team   **Additional PPE**   - 1 medical mask per additional staff member per half day session (2 per full day session) - 1 pair of gloves per additional staff member per half day session (2 per full day session)   **Physical distancing supplies**   - None   **Screening equipment**   - 1 thermometer per vaccination team |
| Routine outreach | **Additional personnel**   - 1 additional member of staff per vaccination team   **Additional PPE**   - 1 medical mask per additional staff member per half day session (2 per full day session)   **Physical distancing supplies**   - None   **Screening equipment**   - None | **Additional personnel**   - 2 additional members of staff per vaccination team   **Additional PPE**   - 1 medical mask per additional staff member per half day session (2 per full day session) - 1 pair of gloves per additional staff member per half day session (2 per full day session)   **Physical distancing supplies**   - None   **Screening equipment**   - 1 thermometer per vaccination team |
| Routine facility-based | **Additional personnel**   - 0.2 additional FTE per facility   **Additional PPE**   - 1 medical mask per additional staff member per half day session (2 per full day session)   **Physical distancing supplies**   - 1 roll of tape per facility per week   **Screening equipment**   - None | **Additional personnel**   - 1.4 additional FTE per facility   **Additional PPE**   - 1 medical mask per additional staff member per half day session (2 per full day session) - 1 pair of gloves per additional staff member per half day session (2 per full day session)   **Physical distancing supplies**   - 1 roll of tape per facility per week - 1 screening tent per facility   **Screening equipment**   - 1 thermometer per facility |

**Scenario 3: Context adjustments.**

| **Strategy** | **Low-intensity scenario** | **High-intensity scenario** |
| --- | --- | --- |
| Campaign | - Assuming an extended duration of the campaign due to a 80% reduction of the daily number of children vaccinated | - Assuming an extended duration of the campaign due to a 50% reduction of the daily number of children vaccinated |
| Outreach | - Half the frequency of sessions | - Double the frequency of sessions |
|  | - Additional outreach to compensate for a drop in facility-based delivery by 25% - Additional outreach to compensate for a drop in school-based delivery by 50% (Indonesia only) | - Additional outreach to compensate for a drop in facility-based delivery by 50% - Additional outreach to compensate for a drop in school-based delivery by 100% (Indonesia only) |

**Scenario 4: Operational cost increases.**

| **Strategy** | **Low-intensity scenario** | **High-intensity scenario** |
| --- | --- | --- |
| Campaign | - An increase of 50% of all cost components potentially affected due to COVID-19 | - An increase of 100% of all cost components potentially affected due to COVID-19 |
| Routine | - Social mobilization: increase by 50% of mean cost | - Social mobilization: increase by 100% of mean cost |
|  | - Training: increase by 50% of mean cost | - Training: increase by 100% of mean cost |
|  | - No hazard pay | - Hazard pay at 20% of salary |

**Table E. Tanzania case study: summary of assumptions by cost category.**

| **Cost category** | **Low-intensity scenario** | **High-intensity scenario** |
| --- | --- | --- |
| **PPE** | - 1 medical mask per health worker per half day session (2 per full day session) - 1 biohazard waste bag per session per outreach team | - 1 medical mask per health worker per half day session (2 per full day session) - Vaccinators receive one set of reusable goggles - 1 pair of gloves used per beneficiary - 1 pair of gloves for non-vaccinator health workers per half day session (2 per full day session) - 1 biohazard waste bag per session/team per outreach team |
| **IPC** | - 12ml of hand sanitizer per beneficiary - Simple hand washing station for each fixed vaccination post (2 x 60-liter bucket) per outreach site - 12ml of soap per beneficiary for outreach sessions | - 12ml of hand sanitizer per beneficiary - Advanced hand washing station for each vaccination post (2 x 60-liter bucket, 2 x stand, 2 x basin) per outreach site - 12ml of soap per beneficiary for outreach sessions |
| **Physical distancing and screening** | - 1 FTE per health facility and 1 additional staff per outreach team (with PPE) - 1 roll of tape per facility per week | - 1 FTE per health facility and 2 additional staff per outreach team (with PPE) - 1 thermometer per vaccination per health facility and per outreach team - 1 roll of tape per facility per week - 1 screening tent per facility |
| **Hazard pay** | - Hazard pay at 10% of base salary | - Hazard pay at 25% of base salary |
| **Training** | - Increase by 50% of mean cost | - Increase by 100% of mean cost |
| **Social mobilization** | - Increase by 50% of mean cost | - Increase by 100% of mean cost |
| **Delivery strategy mix** | - Half the frequency of outreach sessions - Additional outreach to compensate for a drop in facility-based delivery by 25% | - Double the frequency of outreach sessions - Additional outreach to compensate for a drop in facility-based delivery by 50% |
